# Investigating the incidence of youth mental health problem by merging person register and school level class room survey data

**DOI:** 10.23889/ijpds.v1i1.285

**Published:** 2017-04-19

**Authors:** Marko Merikukka, Tiina Ristikari, Reija Paananen, Mika Gissler

**Affiliations:** National Institute for Health and Welfare, Finland; Diaconia University of Applied Sciencies

## Objectives

Aim of the study is to investigate the incidence of youth mental health problems by merging two different types of datasets; a person register, the 1987 Finnish Birth Cohort and school level class room survey data, Finnish School Health Promotion study.

## Approach

The 1987 Finnish Birth Cohort is a longitudinal nationwide follow-up data including a complete census of children born in a single year 1987. Children have been followed subsequently over time from the prenatal period through the year 2012, using official registers collected by Finnish authorities. Register data includes forms of documentation of children's own and their parents' health status and social circumstances from the perinatal period into early adulthood.

Finnish School Health Promotion study is a cross-sectional classroom survey that has been carried out in the Finnish lower secondary schools nationally every other year since 1996. Depending on the geographical location of the school, half of the age group (born in 1987) voluntarily responded to the survey on their health and lifestyle habits in 8th grade (2002) and other half in 9th grade (2003).

The 1987 Birth Cohort data includes a unique numeric code regarding the school the cohort members attended in 2003. The Finnish School Health Promotion study dataset also includes the same unique school code from the year 2003 through which it's possible to merge datasets. The linked data can be analyzed both at school and municipality level.

## Results

There were 59 476 children in the 1987 Finnish Birth Cohort, of whom 57 620 (96.9 %) had school code. School health survey was filled by 48 146 of the 1987 age group. There was positive correlation between self-reported help from home and F-diagnoses F40-F49 (correlation: 0.137, p-value: 0.011). Also self-reported depression score correlated with F-diagnoses F10-F19 (correlation: 0.152, p-value: 0.005) in municipality level.

## Conclusion

It is possible to merge datasets from school level to an individual level register data. The merged big data offers new possibilities to study questions related to the prevalence of mental health problems. The new linked data can be further analyzed to hierarchical model.

